# Ornamental origins and genomic frontiers: a review of big-bracted dogwood research

**DOI:** 10.3389/fpls.2025.1735902

**Published:** 2026-01-30

**Authors:** Trinity P. Hamm, Robert N. Trigiano, Marcin Nowicki, Erin L. P. Moreau, Thomas J. Molnar, Qiu-Yun Jenny Xiang, Sarah L. Boggess, Tarek Hewezi, William E. Klingeman, Denita Hadziabdic, Margaret E. Staton

**Affiliations:** 1 Department of Entomology and Plant Pathology, University of Tennessee, Knoxville, TN, United States; 2 Biosciences Division, Oak Ridge National Laboratory, Oak Ridge, TN, United States; 3 Department of Plant Pathology, University of Minnesota, St. Paul, MN, United States; 4 Department of Plant Biology, Rutgers University, New Brunswick, NJ, United States; 5 Department of Plant and Microbial Biology, North Carolina State University, Raleigh, NC, United States; 6 Department of Plant Sciences, University of Tennessee, Knoxville, TN, United States

**Keywords:** breeding, *Cornus florida*, *Cornus kousa*, *Cornus nuttallii*, cultivars, phylogenetics, population genetics, quantitative genetics

## Abstract

The big-bracted (Benthamidia) dogwood clade consists of small- to medium-sized deciduous trees within the genus *Cornus*, known for their showy spring-time floral bract display. *Cornus* is within the family Cornaceae and order Cornales, and as Cornales is one of the earliest diverging asterids, these taxa have been important for phylogenetic research. Three species within the big-bracted clade, flowering (*Cornus florida*), kousa (*C. kousa*), and Pacific (*C. nuttallii*) dogwoods, are popular ornamental landscape plants in North America, with more than 130 cultivars released. Despite their commercial popularity, numerous research gaps have limited the expansion of fundamental research and dogwood breeding programs. In this present review, we aim to provide a thorough overview of our current understanding of 1) the phylogenetic and biogeographic context, 2) plant biology and major pests and pathogens impacting commercialization, 3) historical commercialization and propagation methods, and 4) genetic and genomic resources and how they have been implemented to understand these species. Research gaps and future directions to advance basic research and breeding of big-bracted ornamental dogwoods are discussed throughout.

## 1 Introduction

Big-bracted (Benthamidia) dogwoods are a clade within the *Cornus* genus, prized for their horticultural use and best known for their large and brightly colored spring-time floral bracts. Three big-bracted species are popular landscape plants and ornamental nursery crops in North America, two of which are native: *Cornus florida* L., flowering dogwood, native to the eastern United States (U.S.), and *C. nuttallii* Audubon ex Torr. & A. Gray, Pacific dogwood, native to the western U.S. The third species, *C. kousa* Hance, kousa dogwood, originates from eastern Asia. In 2019, dogwood species collectively ranked third in value among deciduous flowering trees, in the U.S. Annually, dogwoods generate more than $31 million (USD) in revenue from more than 1 million trees (USDA-NASS, 2020).

The three big-bracted dogwoods commonly used in landscape plantings are deciduous trees or shrubs, with flowering and kousa dogwoods reaching heights of 6–10 meters. In contrast, Pacific dogwood is one of the largest big-bracted dogwoods, reaching 20 meters with an open growth habit (Dirr, 2006). The most characteristic aesthetic feature of cultivated dogwoods is their showy floral bracts, which are modified leaves that subtend a cluster of small true flowers. Although all three commonly have white bracts in their native distributions, flowering dogwood exhibits a range of bract pigmentation across cultivars, from white to pink to red. Kousa dogwood has a more limited range of white to pink bracts available to consumers, whereas cultivated Pacific dogwood has uniformly white bracts. Additional desirable ornamental features of all three dogwoods include brilliant red to dark purple foliage and distinct red or pink berries in the fall.

Flowering dogwood is a cultural icon in the U.S. and is the officially recognized state flower or tree in North Carolina, Virginia, and Missouri. Whereas flowering dogwood makes up the majority of the commercial market share in the U.S., kousa dogwood has gained popularity in the U.S. since the 1990s, particularly in northern zones, due to its increased cold hardiness and pathogen resistance (Ranney et al., 1995; Trigiano et al., 2004). Kousa dogwood also blooms approximately one month later than flowering dogwood, and the floral display lasts longer (Cappiello and Shadow, 2005). Pacific dogwood is typically grown as an ornamental plant mainly within its native range due to its limited winter hardiness and heat tolerance (Cappiello and Shadow, 2005).

**Figure 1:**
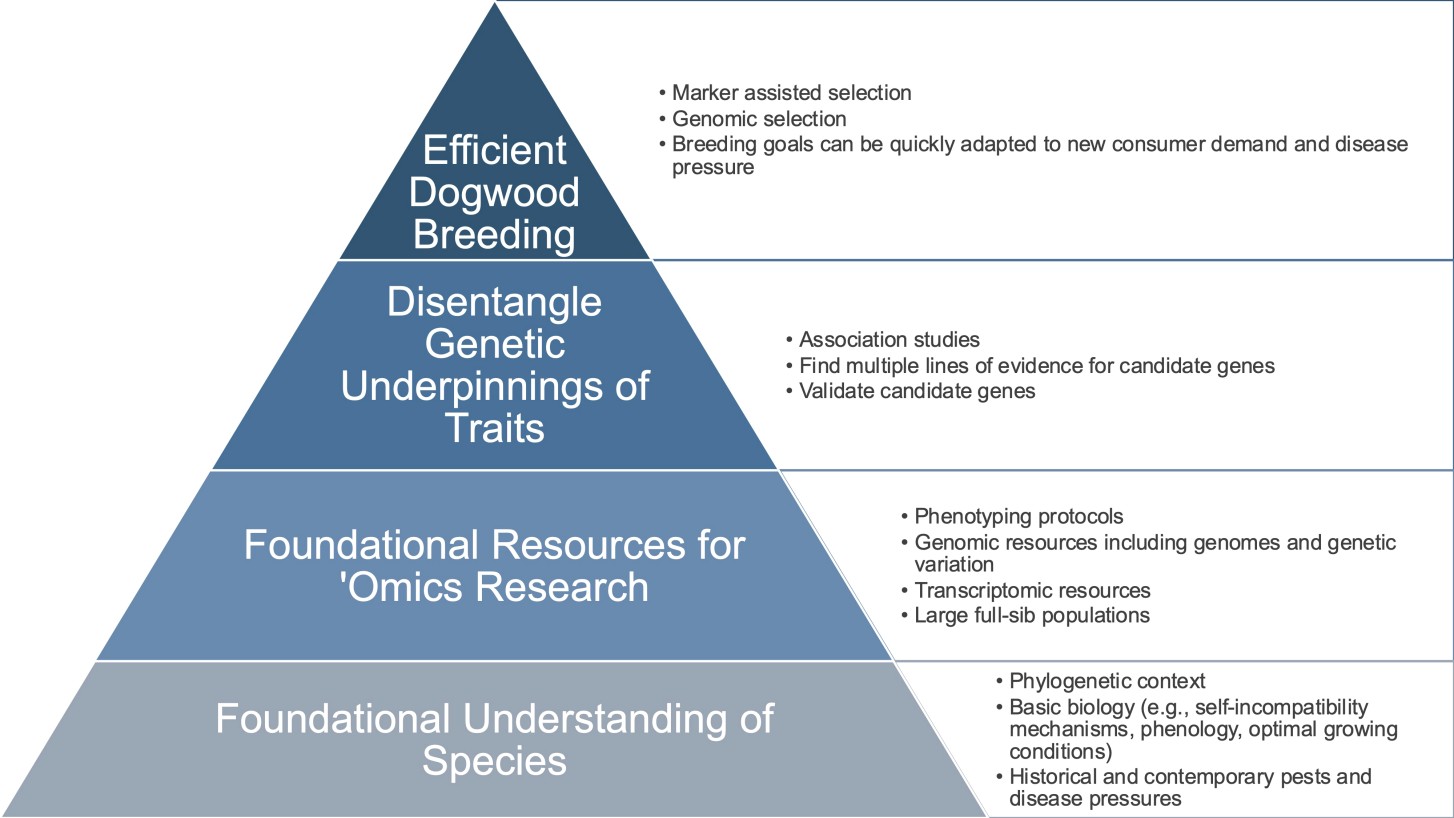
Efficient dogwood breeding conceptual framework from understanding the species to genetic underpinnings of traits of interest.

Despite the economic importance of dogwoods, there is only one active public breeding program in the U.S, the Rutgers University Woody Ornamentals Breeding Program. Currently, their primary focus includes flowering and kousa dogwoods, however, Pacific dogwoods were historically used in the breeding program to create interspecific hybrids, and their genetic contributions remain in the active breeding pool (Moreau et al., 2024). The breeding goal for flowering dogwood is to combine red and pink colored bracts with pathogen tolerance, improved bloom displays, and enhanced growth and architecture. For kousa dogwood, where resistance and tolerance to pathogens are commonplace in the species, the breeding efforts focus on producing more stable and deeper pink bract coloration, larger and unique bract shapes, and extended bloom times (Molnar, 2022). These efforts are guided by observational phenotyping, with the recent emergence of genetic and genomic resources that can be leveraged to inform breeding (Pfarr Moreau et al., 2022; Moreau et al., 2024; Hamm et al., 2025). With a 3- to 5-year generation time, breeding and phenotyping are limited by both time and space constraints (Cappiello and Shadow, 2005), which suggests that predictive genetic and genomic knowledge and tools could help increase breeding efficiency (**Figure 1**).

While the majority of research on big-bracted dogwood has focused on its ornamental and aesthetic aspects for landscape use, the genetics, genomics, and evolutionary biology of the genus have also been explored. Genetic markers have been developed for flowering and kousa dogwoods and have been utilized in population genetics (Hadziabdic et al., 2010, Hadziabdic et al., 2012; Mitchell et al., 2023), quantitative trait loci (QTL) (Wadl et al., 2011; Parikh et al., 2017; Pfarr Moreau et al., 2022), and cultivar identification studies (Windham and Trigiano, 1998; Trigiano et al., 2004; Smith et al., 2007). Transcriptomes (Zhang et al., 2013; Yu et al., 2017; Melton et al., 2020) and a fragmented genome assembly (Bewick and Leebens-Mack, 2019) have also been developed for mainly flowering dogwood. Recently, annotated, diploid genome assemblies were completed for two flowering dogwood cultivars (Hamm et al., 2025). However, historically, without the greater context of a high-quality genome and annotation, it has remained challenging to produce deliverables for breeding programs or fundamentally understand the genetics that drive economically important phenotypic traits. These emerging genomic resources will be essential for progressing dogwood research.

## 2 Phylogenetics and biogeography

The genus *Cornus* contains four major clades: blue- or white-fruited dogwoods, Cornelian cherries, dwarf dogwoods, and big-bracted dogwoods. The big-bracted dogwoods comprise approximately 10 species and eight subspecies out of the 55–65 species (Du et al., 2023). The species are divided into two geographically isolated subclades: *Syncarpea* is found in eastern Asia, whereas *Cynoxylon* is distributed in North America, extending to Central America. Kousa dogwood is a member of the Asian subclade (Subg. *Syncarpea* or clade *Syncarpea*) (Xiang et al., 2006; Du et al., 2024a), and Pacific and flowering dogwoods are members of the American subclade (Subg. *Cynoxylon* or clade *Cynoxylon*) that also contains *C. disciflora* occurring in Mexico to Central America.

The big-bracted dogwoods were consistently recognized as the sister of the dwarf dogwoods (bunchberries) (Subg. *Arctocrania* or Clade *Arctocrania*) in phylogenetic studies of *Cornus*, Cornaceae, and Cornales from analyses of a few or several genes (Xiang et al., 1998, Xiang et al., 2006, Xiang et al., 2011; Xiang and Thomas, 2008) to genome-wide markers (Xiang et al., 1996; Fu et al., 2019; Du et al., 2023) from both plastid and nuclear genomes. An exception was reported in a study of Cornales using Angiosperms353 gene data, where the big-bracted dogwoods were suggested to be sister to the Cornelian cherries, with the dwarf dogwoods diverging first among the four major lineages (Thomas et al., 2021). However, this result is disputed due to the non-optimal alignments of DNA sequences in the dwarf dogwood samples presented in Thomas et al. (2021) (see Du et al., 2023).

The robust phylogenies supported by new, more dense genome-wide DNA sequence markers led to an updated classification of *Cornus* (Du et al., 2023). This update followed the PhyloCode (International Code of Phylogenetic Nomenclature), which only assigns names to clades without a taxonomic rank (Cantino and De Queiroz, 2020). In this proposed reclassification scheme, the big-bracted dogwoods are renamed *Benthamidia* and subdivided into *Syncarpea* (the Asian big-bracted species with compound drupes) and *Cynoxylon* (the American big-bracted species with simple drupes) (Du et al., 2023, Du et al., 2024b).

Recent phylogenetic analyses using genome-wide DNA sequence data confirm the *Cynoxylon* clade as sister to the Asian big-bracted dogwoods (*Syncarpea* clade). The monophyly of *Cynoxylon* as a clade was not well supported in early studies, nor by recent plastid DNA sequences (Fu et al., 2019; Du et al., 2023). These studies showed the *Cynoxylon* clade is paraphyletic, and that the subclade flowering dogwood*-C. disciflora* is more closely related to the Asian *Syncarpea* clade than to the Pacific dogwood. This suggested a possibility of ancient hybridization between the ancestor of the *Syncarpea* clade and the ancestor of the flowering dogwood*-C. disciflora*, thereby leading to the capture of the Asian chloroplast genome in the common ancestor of flowering dogwood*-C. disciflora*. Yet, statistical testing with the D-statistic (ABBA-BABA) analysis did not support ancient gene introgression (Du et al., 2023). Short estimated time intervals support the rapid diversification of the big-bracted clade in its early evolutionary history (Du et al., 2023, Du et al., 2024b). Thus, rapid diversification events have left marks of incomplete lineage sorting (ILS) or differential lineage sorting of a dimorphic ancestral plastid genome, which explains the conflicting relationships between the nuclear and plastid phylogenies, as discussed in Du et al (2023, Du et al., 2024b, Du et al., 2024a).

The geographic history of the big-bracted dogwoods has been inferred in recent biogeographic analyses of all *Cornus* or only the *Benthamidia* clade, using dated phylogenies reconstructed from genome-wide data and fossils that contain known living and fossil species (Du et al., 2023, Du et al., 2024a). These analyses suggested a divergence of the big-bracted and dwarf dogwoods in eastern Asia in the very late Cretaceous (~70 mya), each subsequently spreading. The ancestor of the big-bracted dogwood clade inhabited a trans-Beringia range in Eurasia and western North America, including Mexico, up to the late Oligocene (~28 mya), before diverging into the eastern Asian *Syncarpea* and *Cynoxylon* clades. The extinction of the *Cynoxylon* clade in Europe and eastern Asia resulted in the present-day geographic isolation of the two groups (Figure 7. in Du et al., 2023; Figure 6 in Du et al., 2024a). An ancestor of the *Cynoxylon* clade diversified in western North America during the Miocene (~23 mya), resulting in Pacific dogwood in the west, *C. disciflora* in the south, and flowering dogwood in the east of its range. Flowering dogwood then later spread into eastern North America from Mexico, forming two distinct subspecies. Therefore, within America, the most recent common ancestor of the big-bracted dogwoods likely first occurred in western North America before expanding into Mexico, Central America, and eastern North America. During the mid-Miocene and Pliocene (5–13 mya), two episodes of rapid diversification occurred in the eastern Asia *Syncarpea* clade (Du et al., 2024a). A speciation event in the mid-Miocene (~14 mya) resulted in the separation of the temperate/northern kousa dogwood and the subtropical/southern *C. hongkongensis* complex (Du et al., 2023).

**Figure 2:**
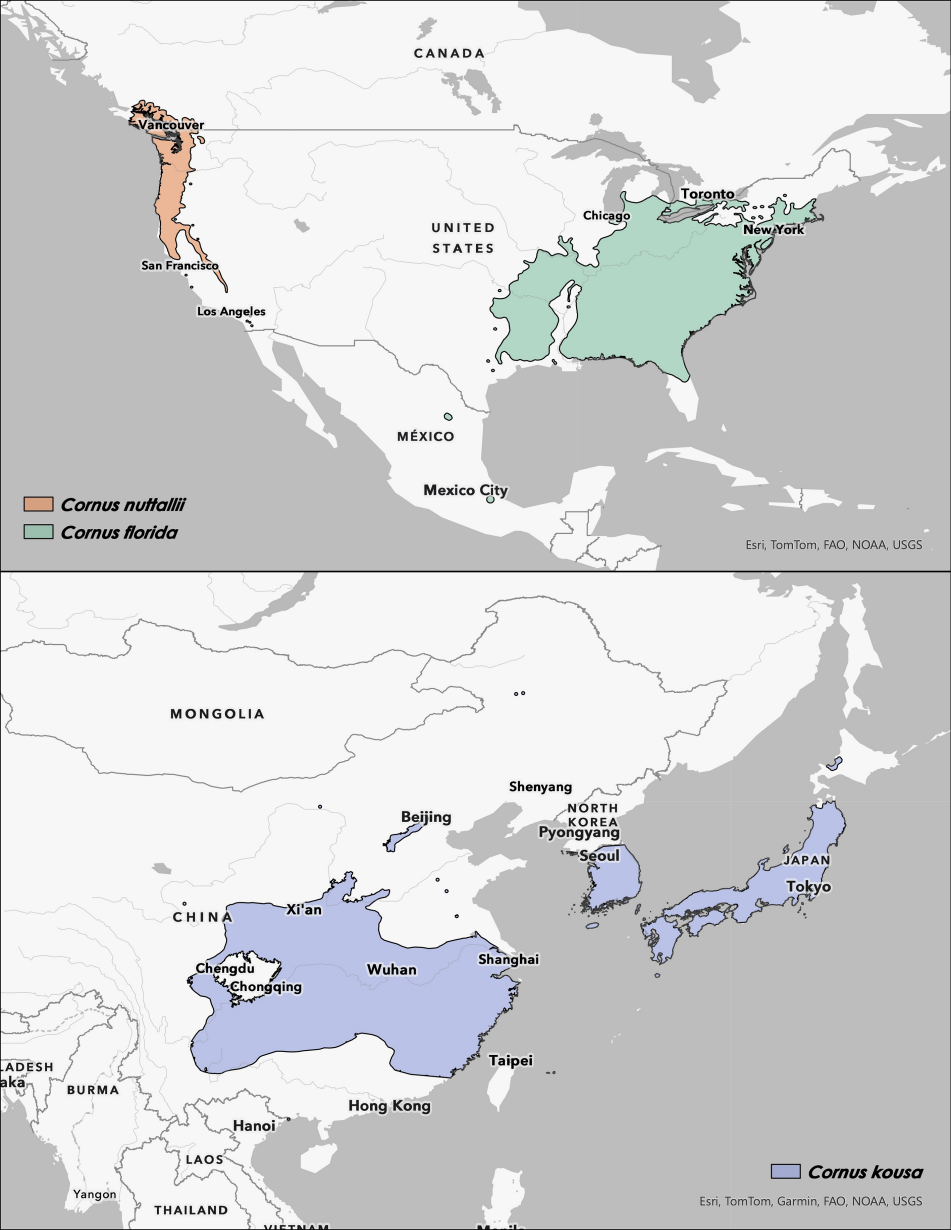
Species range maps *Cornus florida*, *C. nuttallii*, and *C. kousa* (Map Source: Esri, TomTom, Garmin, FAO, NOAA, USGS). **(A)***Cornus florida* (green) is native to eastern North America (Data Source: Thompson, 2011), while *C. nuttallii* (orange) is restricted to western North America (Data Source: PlantMaps, 2025) (Map Extent: 1:40,000,000). **(B)***Cornus kousa* (blue-grey) is a wide-ranging Asian species found natively throughout China, Japan, Korea, and surrounding islands (Map Extent: 1:30,000,000). The *C. kousa* range map was derived from GBIF occurrences based on human observations and preserved specimen collection localities (doi: 10.15468/DL.SRHFN5). Additional methods available in **Supplementary File 1**.

**Figure 3:**
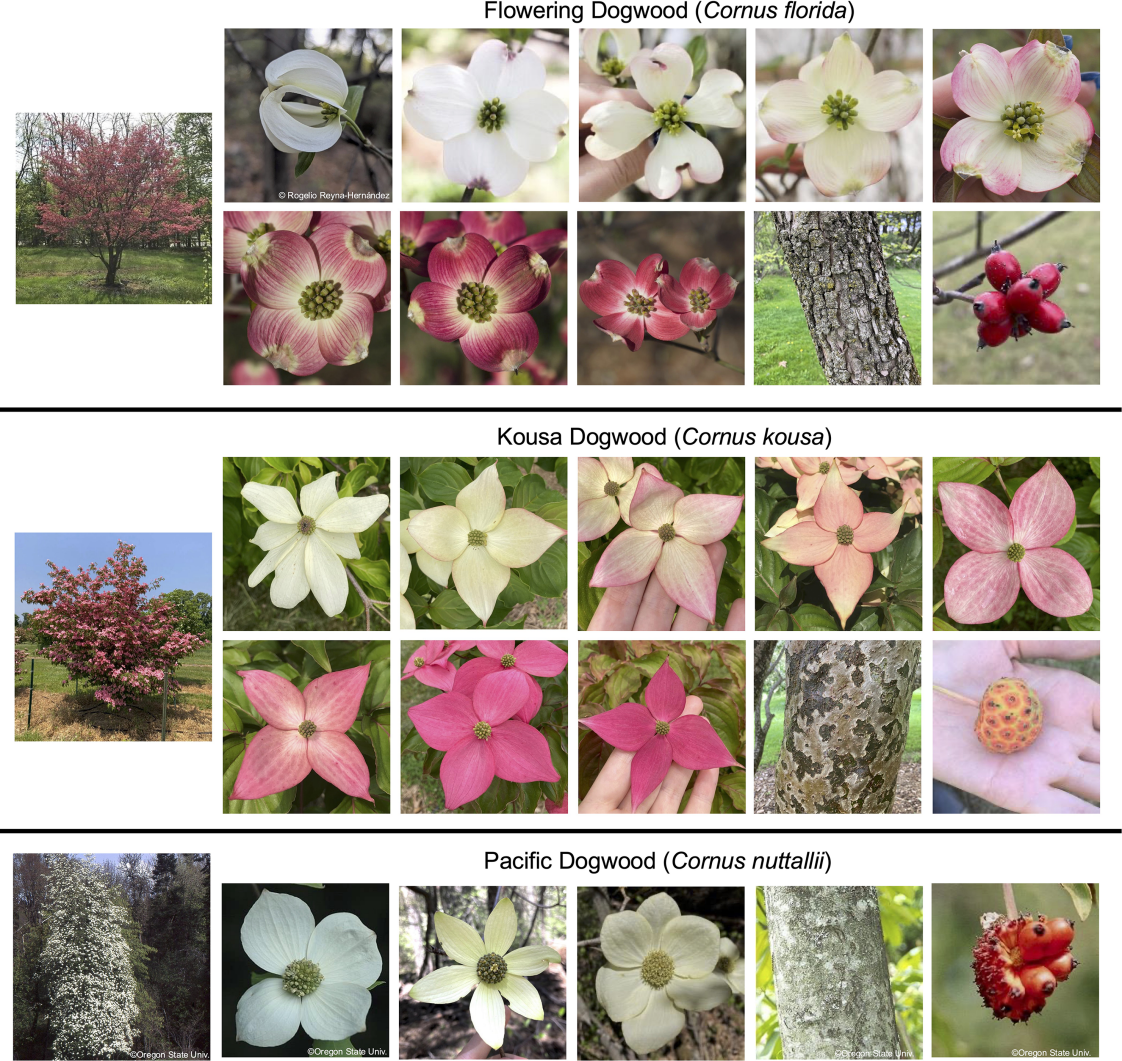
Big-bracted dogwood (*Cornus* spp.) flowering diversity. Full-flowering trees are shown on the left. Bract, fruit, and bark variation across flowering dogwoods are on the top, kousa dogwood in the middle, and Pacific dogwood on the bottom. (*Cornus florida* subsp. *urbinana*^©^https://creativecommons.org/licenses/by-sa/4.0/).

Flowering dogwood is found throughout much of the southeastern U.S. and as far north as Maine (**Figure 2A**; map construction methods available in **Supplementary File 1**). As recently as the Last Glacial Maximum (~21 kya), it had a predicted continuous distribution much further south, but now only small relict populations remain in high elevation mountain ranges in Mexico (**Figure 2A**) (Call et al., 2016). The future distribution of flowering dogwood was predicted to expand north but shrink in the Midwest of the U.S. under a global warming scenario (Call et al., 2016). The flowering dogwood endemic to Mexico is a unique subspecies, *C. florida* subsp. *urbiniana*, separate from the main native range with *C. florida* subsp. *florida*. The four bracts of *C. florida* subsp. *urbiniana* are narrow and not separate at the tips, forming a lantern-like structure, distinct from the bract morphology in typical flowering dogwood (**Figure 3**) (Dudley and Santamour, 1992). This difference in the bracts of *C. florida* subsp. *urbiniana* is consistent, independent of where it is grown (Dudley and Santamour, 1992). In addition to the two subspecies, there is also *C. florida* forma *rubra*, characterized by its pink bracts.

The Pacific dogwood occupies a much smaller range than flowering dogwood in the east. It occurs primarily in the Pacific Northwest, west of the Cascade and Sierra Nevada mountain ranges, extending south from southern British Columbia through Washington, Idaho, Oregon, and California (**Figure 2A**). The kousa dogwood currently occupies a natural distribution in eastern and central China, extending to Korea and Japan (**Figure 2B**). It also has two subspecies that are geographically separated, with subsp. *chinensis*, native to China, and subsp. *kousa*, native to Japan and Korea. In contrast to the two subspecies of flowering dogwood, these subspecies of kousa dogwoods are somewhat difficult to differentiate phenotypically (Rehder, 1927; Cappiello and Shadow, 2005), with noted differences in leaf texture and color, as well as the morphology of inflorescence peduncles (Xiang, 1987).

## 3 Plant biology

### 3.1 Flowering dogwood (*C. florida*)

In addition to its horticultural importance, flowering dogwood is a common understory tree found in many coniferous and deciduous forests throughout eastern North America, where it plays crucial ecological roles. Flowering dogwoods are pollinated by a variety of insects, including andrenid bees, halictid bees, and cerambycid beetles (Rhoades et al., 2011). Various mammals and birds also utilize the flowering dogwood as a food source and habitat (Stiles, 1980; Eyde, 1988). Many tissues of the flowering dogwood are high in calcium, with the deciduous leaves playing a crucial role in maintaining calcium in circulation within the upper bioactive layers of the soil (Thomas, 1969). The fruits are an important source of nutrients for wildlife due to their high levels of calcium, fat, and protein (Halls, 1977). The fruits of the flowering dogwood are small, usually single-seeded drupes. The individual drupes develop in clusters of up to 12 and can range in color from orange to red (Cappiello and Shadow, 2005). Flowering dogwoods are also an obligate outcrossing species with gametophytic self-incompatibility where pollen tube growth is restricted (Reed, 2004).

Blooming in late spring, the showy bracts of flowering dogwoods create a striking display in the landscape. Four petaloid bracts with a cleft at the apex subtend and protect the true flowers throughout the winter (Dirr, 2006). Once bud break occurs, blooms are susceptible to frost damage for this early-flowering tree. While generally white throughout wild populations, the color of the bracts can vary in subsp. *florida* (see *Bract and Foliar Coloration* section for more information; **Figure 3**). Although also usually white, “soft pink” bracts have been noted in a population of subsp. *urbiniana* near Ciudad Victoria in Tamaulipas State, Mexico (Dudley and Santamour, 1992), but are not cultivated. For subsp. *florida*, the bracts can be separate, overlapping, or even supernumerary. The leaves typically emerge only after peak flowering. However, phenology varies among cultivars and individuals and depends on air temperature (Reader, 1975).

Whereas the petaloid bracts are the most striking ornamental feature of flowering dogwoods, several additional traits contribute to their appeal as a four-season ornamental tree. For cultivars with pink or red bracts, the first flush of leaves in the spring is also red, with the color fading to green by summer. Cultivars with variegated leaves are also available (Cappiello and Shadow, 2005). Beyond the basic rounded canopy of flowering dogwoods, cultivars are available with columnar, dwarf, or weeping architectures (Dirr, 2006). In the fall, all flowering dogwoods develop bright red foliage and red drupes. If not eaten by wildlife or abscised, drupes will persist into November and December after the leaves have senesced, with the stark red display of drupes complementing the graceful architecture of the tree (Cappiello and Shadow, 2005).

### 3.2 Kousa dogwood (*C. kousa*)

Kousa dogwood is also an understory tree and is found in heterogeneous habitats throughout its native range (Yuan et al., 2019). In its native range, kousa dogwoods are pollinated by bees and butterflies (Yuan et al., 2019). Although not native to the U.S., kousa dogwoods can be pollinated by insects found in eastern Tennessee, including halictid bees, scarab beetles, cerambycid beetles, and cantharid beetles (Rhoades et al., 2011). However, it remains unclear which of these insects serves as the most efficient pollinator, and to what extent this varies based on the geographical distribution of the pollinators. Kousa dogwoods have fleshy, aggregate fruits (drupes) that range in color from red to orange to pink and are edible if eaten fresh. In China and Korea, the fruits are eaten and dispersed by birds (Yuan et al., 2019) and also used to produce wine (Du et al., 1974; Vareed et al., 2006). Although the specific mechanism is unknown in kousa dogwoods, they are self-incompatible just like flowering dogwoods (Wadl et al., 2009).

The species is especially popular as a landscape ornamental plant in the northern U.S. for its cold hardiness, with subsp. *chinensis* generally regarded as the relatively hardier subspecies (Cappiello and Shadow, 2005). The general ornamental traits of kousa dogwoods are similar to those of flowering dogwoods, with a four-season appeal. However, there are several key differences in their biology. The leaves of kousa dogwoods emerge before the trees bloom, and flowering occurs about a month later compared to flowering dogwoods, regardless of the growing zone, thus better protecting the blooms from late frosts. The bracts of kousa dogwoods may be separate, overlapping at the base, or rarely fused (Wadl et al., 2014). Kousa dogwood bracts consistently have pointed tips that are distinct from those of flowering dogwood (**Figure 3**). The floral display of kousa dogwood also lasts longer than flowering dogwood. However, there are fewer color options, and the intensity of color is more dependent upon temperature and the environment (Cappiello and Shadow, 2005; Molnar et al., 2017; Molnar, 2022). Fall foliage can be striking depending on the cultivar, and the majority of cultivars exhibit exfoliating bark that is a patchwork of gray, copper, and olive green (Dirr, 2006). The growth habit of kousa dogwoods is generally more vase-shaped and/or bush-like, especially as a young tree, which can help distinguish them from flowering dogwoods when inflorescences or fruits are not visible. Kousa dogwoods also have cultivars with weeping, dwarf, and narrow growth forms and variegated leaves (Cappiello and Shadow, 2005).

### 3.3 Pacific dogwood (*C. nuttallii*)

Pacific dogwood, found primarily on the western coast of North America, is notable for its large, springtime bracts that resemble those of flowering dogwood but are typically larger and more rounded (**Figure 3**; Cappiello and Shadow, 2005; Dudley and Santamour, 1992). Unlike flowering dogwood, Pacific dogwood tends to grow in more shaded environments and is often found in riparian zones or mixed coniferous forests (Thomas, 1969). It thrives in moist, well-drained soils, typically at low to mid-elevations. The tree prefers partial shade but can tolerate full sun in cooler climates, and it is well-adapted to the mild, wet winters and dry summers typical of the Pacific Northwest. The fruits of Pacific dogwood are also drupes, develop in clusters, and serve as an important food source for various wildlife species. However, they are generally larger than those of flowering dogwood (Halls, 1977). Similar to flowering and kousa dogwoods, Pacific dogwood is insect-pollinated and an obligate outcrossing species (Keir et al., 2011).

Pacific dogwood blooms in spring, and its flowering period can be influenced by local climate conditions (Reader, 1975). The leaves of Pacific dogwood typically emerge after flowering, and the tree exhibits a similar four-season appeal with attractive fall foliage and persistent drupes (Cappiello and Shadow, 2005). The bark of mature trees is smooth and gray, often developing a patchwork appearance as it exfoliates, similar to kousa dogwood. The leaves are simple, opposite, and deciduous, turning a vibrant red in the fall, which adds to the tree’s ornamental appeal (Cappiello and Shadow, 2005). Even though Pacific dogwood shares many ornamental traits with flowering and kousa dogwoods, its larger size, susceptibility to the dogwood anthracnose pathogen, preference for specific environmental conditions, and limited cold hardiness make it less commonly cultivated (Dirr, 2006). Despite these challenges, it remains a sought-after species for gardens and parks in its native range, where its beauty and ecological value can be fully appreciated.

### 3.4 Pests and pathogens

Over the past three decades, several pathogens have threatened flowering and Pacific dogwoods but on different timelines: dogwood anthracnose, primarily a historical problem; powdery mildew, a disease of current importance; and vascular streak dieback, an emerging disease of high concern. Beyond these three, other pests and pathogens cause comparably more limited damage to dogwoods.

Dogwood anthracnose, caused by *Discula destructiva* Redlin, emerged in the mid-1970s and devastated natural populations of flowering dogwood (Redlin, 1991; Hadziabdic et al., 2010; Pais and Whetten, 2020; Mitchell et al., 2023). Dogwood anthracnose causes branch dieback, necrotic spots and blight on the leaves, and stem cankers, leading to tree death in two to five years (Daughtrey et al., 1988). During infection and before the tree dies, there is also a decrease in fruit production, thereby decreasing food availability to wildlife (Rossell et al., 2001). There were two postulated introductions of dogwood anthracnose to the U.S., one on the western coast, where the fungal plant pathogen mainly infected Pacific dogwood, and one on the eastern coast, where it infected flowering dogwood (Mantooth et al., 2017). Kousa dogwoods vary in levels of tolerance to *D. destructiva* and are more tolerant than flowering or Pacific dogwoods (Santamour et al., 1989; Ranney et al., 1995). To aid in understanding this disease, new telomere-to-telomere genomic resources have recently been released for *D. destructiva* (Niece et al., 2025). Infections are still being monitored in Canada today (Elmhirst, 2024), but not in the U.S. Despite its historic impact on North American forests, dogwood anthracnose is currently not a major issue in forests or in nursery production. It is hypothesized that in forests, the most susceptible individuals have already died (Daughtrey, 2014). Whereas in nurseries it is mainly controlled by an aggressive spray schedule and the introduction of naturally resistant cultivars (Fulcher et al., 2012).

The main contemporary issue for nursery-grown flowering dogwoods is powdery mildew caused by the fungus *Erysiphe pulchra* (Cooke & Peck) U. Braun & S. Takam (Klein et al., 1998). This disease emerged in the U.S. in 1994 and has led to millions of dogwoods losing their commercial value (Li et al., 2009). Although only fatal to seedlings, powdery mildew causes leaves to curl and distort, and can decrease flowering and growth over time, rendering infected trees unmarketable (Li et al., 2009). The disease was first reported on flowering dogwood in 1887 (Burrill and Earle, 1884), however, it was rarely reported in the U.S. until 1994 (Hagan et al., 1998). In 1994, powdery mildew was reported simultaneously in the forests, landscapes, and nursery plantings of flowering dogwood in Alabama, Florida, and Georgia (Hagan et al., 1998). Low genetic diversity, limited population structure, and high linkage disequilibrium have been observed in *E. pulchra* populations across the U.S (Wyman et al., 2019). These results suggest the epidemic was due to a recent introduction, and the predominant mode of reproduction is through asexual conidia. A draft genome is available for *E. pulchra* (Wadl et al., 2019).

Powdery mildew has dramatically impacted the dogwood industry, resulting in increased management costs for producers (Li et al., 2009). According to the most recent estimate, nurseries in Tennessee, U.S., are spending approximately $938/ha/year (USD) on foliar pathogen management for dogwoods (Liyanage et al., 2024). To control the pathogen, fungicide applications must be applied every two weeks from May to October. This intensive regimen led many small-scale growers to cease production due to the high costs of materials and labor (Li et al., 2009; Fulcher et al., 2012). Improved cultural practices, such as increasing plant spacing to maximize airflow among trees, can help decrease disease severity, however, they do not provide complete control (Fulcher et al., 2012).

Although substantial efforts have been made to identify flowering dogwood cultivars with resistance (i.e., actively and successfully reduces pathogen levels) or tolerance (i.e., active pathogen infection but little to no symptoms of disease), naturally occurring resistance or tolerance in these trees is very rare (Windham and Witte, 1998). More than 20,000 seedlings were screened for *E. pulchra* tolerance in an abandoned dogwood nursery field in Tennessee, U.S., and only four tolerant seedlings were identified and eventually marketed as powdery mildew-tolerant cultivars (Windham et al., 2003; Trigiano et al., 2016). Targeted breeding of flowering dogwood for pathogen tolerance has proven challenging due to the long generation times, self-incompatibility, inbreeding depression, and variable pathogen pressure, which is likely influenced by environmental factors (Molnar, 2018). As a result, kousa dogwoods have gained increased popularity in the U.S. for their greater disease tolerance compared to flowering or Pacific dogwoods (Ranney et al., 1995; Windham et al., 2005). The majority of kousa dogwood cultivars that were already on the market were naturally resistant to both powdery mildew and dogwood anthracnose (Santamour et al., 1989; Ranney et al., 1995; Windham et al., 2005; Li et al., 2007). For flowering dogwood, research has shifted to molecular and genetic approaches to identify the mechanisms underlying powdery mildew tolerance (see the *QTL* and *RNA-sequencing* sections below).

Vascular streak dieback (VSD) is an emerging malady of many tree species and has been mainly reported in nursery stock, including both flowering and kousa dogwoods (Adams, 2025; Bily et al., 2025b; Liyanapathiranage et al., 2025). Symptoms include stunting, leaf chlorosis, wilt, dieback, and eventual death. This has resulted in large losses of nursery stock, with a severe economic impact on the nursery industry in some southern states (Liyanapathiranage et al., 2025). Although Koch’s postulates have not been fulfilled for any of the affected plant species, recent evidence suggests that *Ceratobasidium* sp. is the causal agent (Bily et al., 2025a; Liyanapathiranage et al., 2025). In polymerase chain reaction (PCR) assays, *Ceratobasidium* sp. has been detected in only 28.6% of the samples tested, and therefore, additional research is needed to improve diagnostic tools (Bily et al., 2025a). Additionally, there are currently no effective preventive or curative treatments (Liyanapathiranage et al., 2025). Understanding of this disease complex is currently limited, and research and cooperation are needed to develop effective management strategies.

Other pests and pathogens that impact dogwoods in the U.S. are the dogwood borer, several ambrosia beetle species, scale insects, and spot anthracnose (Fulcher et al., 2012). The common dogwood borer (*Synanthedon scitula*) (Harris, 1839) is the most important dogwood pest but is not economically important. The common dogwood borer can be mitigated with cultural techniques such as timely pruning and avoiding physical injury to the tree (Fulcher et al., 2012). Spot anthracnose, caused by *Elsinoë corni* Jenkins and Bitanc., is a common but not economically important disease. The pathogen produces red to purple, elliptical to circular lesions on the young and emerging leaves and bracts but is primarily cosmetic and most prevalent when trees are in full sun (Hagan et al., 1998).

### 3.5 Bract and foliar coloration

Beyond pathogen resistance, the springtime color of the bracts is the second most economically important trait of dogwoods. The bract color of flowering dogwoods can vary from white to pink to deep red, with diverse pigmentation patterns concentrated in the veins, apices, sides, or spread throughout the bract. In contrast, the majority of kousa dogwood cultivars exhibit white-to-cream colored bracts, although some are varying degrees of pink (**Figure 3**) (Cappiello and Shadow, 2005). The first flush of leaves on flowering and kousa dogwood cultivars varies in color, with some cultivars having green leaves and others having red-pigmented leaves. The anthocyanin biosynthetic pathway regulates these pink and red colors in the vegetative tissues of dogwoods (Wadl et al., 2011; Hamm et al., 2025; Pavlović et al., 2025).

Although anthocyanin pigment biosynthesis is well-studied in plants, research on this process in dogwoods, especially at the cultivar level, remains preliminary. Anthocyanin profiling in ripe fruits and leaves of kousa dogwoods reveals that cyanidin-3-glucoside is the predominant anthocyanin, followed by delphinidin-3-glucoside, cyanidin-3-galactoside, and trace amounts of pelargonidin-3-glucoside (Du et al., 1974; Vareed et al., 2006; Khan et al., 2018). In the fruits of flowering dogwood, the cyanidin-3-galactoside and cyanidin-3-glucoside are the most commonly identified anthocyanins, followed by smaller amounts of delphinidin-3-glucoside and delphinidin-3-galactoside, and trace amounts of pelargonidin-3-galactoside and cyanidin-3-arabinoside (Du et al., 1974; Vareed et al., 2006). Recently, anthocyanin profiling has been completed on flowering dogwood leaves and bracts, and the primary anthocyanins detected in both tissues were delphinidin 3-O-galactoside and delphinidin 3-O-sambubioside (Hamm et al., 2025). Therefore, the kousa dogwood bracts remain the only tissue not profiled for anthocyanin compounds contributing to the pigmentation.

Our understanding of the genetic and environmental regulation of anthocyanin production in dogwoods has historically been based on observations from breeding programs. The color intensity of bracts and leaves has been observed in the Rutgers University Woody Ornamentals Breeding Program for both flowering and kousa dogwood. Light, temperature, and disease impact bract and leaf color for both species (Orton, 1982; Molnar, 2022). For example, pink-bracted dogwoods exhibit the most saturated bract color in the spring with cool, wet weather during bract development. However, white flowering dogwood bracts can turn mottled red/pink due to frost damage or spot anthracnose infection. The first flush of leaves in flowering dogwood is usually (but not always) indicative of the bract color, thereby suggesting that the genes regulating leaf and bract color may be tightly linked or possibly the same (Orton, 1982). In contrast, the advanced interspecific hybrids (see *Breeding History* section for more information on breeding strategy) within the Rutgers Breeding Program can vary regarding this trait, and bract color is not always consistent with the color of the first flush of leaves. In kousa dogwood, the deepest pink cultivar to be released, Scarlet Fire^®^ (Molnar et al., 2017), is actually an advanced interspecific hybrid, and it has been hypothesized that Pacific dogwood genes could be contributing to the intensity of the pigmentation (see *Population-Level Genetics* section for more information) (Moreau et al., 2024).

Limited quantitative studies have focused on flowering dogwood. From inheritance studies, it was estimated that two QTL (*rl1* and *rl2*) regulate the leaf color of flowering dogwoods (Wadl et al., 2010b), but no stable QTL were detected (Wadl et al., 2011). From the same population, but with updated genomics resources, one highly significant QTL was found to control the binary presence/absence of red colored bracts/leaves versus white bracts/green leaves (Hamm et al., 2025).

Overall, we have gained an understanding of the mechanisms controlling whether anthocyanins can be accumulated in flowering dogwood. Further research is needed to pinpoint the hypothesized modifier loci that regulate the varying intensities of pigmentation present in flowering dogwoods. Likewise, research is required to identify mechanisms controlling pigmentation in kousa dogwoods and the advanced interspecific dogwoods. Refer to the *QTL*, *Population-Level Genetics*, and *RNA-Sequencing* sections below for further research and progress on genetic controls.

## 4 Ornamental specimen and landscape uses

### 4.1 U.S. crop commodity valuation of dogwood

Within deciduous flowering trees grown as specialty horticultural crops, “dogwood” species are evaluated in aggregate by the U.S. Department of Agriculture within the National Agricultural Statistics Service’s periodic *Census of Horticultural Specialties*. Flowering and kousa dogwood, however, are expected to comprise most of the inventory and sales that were reported by 987 commercial growers in the most recent survey. Nationally, producers reported sales figures for 1.18 million dogwood trees valued at $31.02 million (USD). The majority is from wholesale, with 620 operations reporting $27.89 million (USD) from 1.11 million trees, with the remaining from retail sales for 420 operations, yielding $3.13 million (USD) from annual sales of almost 70,300 trees (USDA-NASS, 2020).

### 4.2 Breeding history

Combining economically important traits in dogwoods has proven difficult, and much of the work remains in its infancy. For example, one of the current main goals for flowering dogwood breeding is to combine red or deep pink bract color with powdery mildew resistance. However, before any breeding could occur, tolerance and/or resistance to powdery mildew had to be identified in flowering dogwood source populations, which has only been done recently (Mmbaga and Sauvé, 2007; Trigiano et al., 2016; Pfarr Moreau et al., 2022). Additionally, breeding programs must overcome relatively long generation times as well as the expense and access to land resources for growing and evaluating thousands of trees to blooming age. The small-scale breeding programs also must balance resources between many small populations for advanced cultivar development and few large full-sibling populations for research purposes. All of these difficulties are further constrained by self-incompatibility and inbreeding depression. As a result, most commercially available cultivars have been selected from chance seedlings by nursery professionals or hobby breeders and have not been explicitly bred following a targeted improvement scheme (Cappiello and Shadow, 2005). When a new cultivar is discovered or created, it is typically patented and/or trademarked, and then asexually propagated via chip budding for distribution to consumers. For more information on propagation, see the sections below.

Pink-red bracts have been a trait desired by consumers for both flowering and kousa dogwood for many years. Pink-bracted flowering dogwood (*C. florida* forma *rubra*) was first documented by Mark Catesby in 1731, after he discovered a single specimen in Virginia and transplanted it, marking the beginning of its horticultural use (Catesby, 1731). Despite the long history, there was little genetic diversity represented in the pink-bracted cultivars for both flowering and kousa dogwood in the 1980s (Orton, 1982; Moreau et al., 2024). The pink-bracted flowering dogwood cultivars available in the 1980s were from only four sources: a wooded area in Knoxville, Tennessee; a large planting of dogwoods on an “estate in the East;” the wild in Alabama; and a pasture in Pennsylvania (Orton, 1982). All of the pink-bracted kousa dogwoods were either from the wild in Japan (*C. kousa* ‘Beni Fuji’ PP8,676), a Japanese origin cultivar ‘Satomi’ (with many synonymous cultivars, e.g., ‘Rosabella’), or bred from these two accessions in the Rutgers Woody Ornamentals Breeding program (Moreau et al., 2024).

Since their emergence, pathogen tolerance to powdery mildew and dogwood anthracnose has also been a breeding focus. Consumer horticultural marketing research has suggested consumers are willing to pay a premium for powdery mildew-tolerant trees, as much as $11.87 (USD) to $16.38 (USD) more per tree (Klingeman et al., 2004). Consumer demand for powdery mildew-tolerant dogwood trees provides a basis for increasing commercial nursery stocks of tolerant flowering and kousa dogwood plant materials, while also contributing to reduced production and tree maintenance costs for fungicide applications during the production cycle (Gardner et al., 2003).

A breeding and research program was established at the University of Tennessee Institute of Agriculture (UTIA) in the 1990s to address these disease pressures (Witte et al., 1999; Wadl et al., 2011). The ‘Appalachian Series’ has since been released for flowering dogwood (**Supplementary File 2**). All the cultivars in the Appalachian Series were originally found either in nature or abandoned nursery fields plagued with powdery mildew. Kousa dogwood seeds were donated to the program by Polly Hill of Vineyard Haven in 1989 and phenotyped for interesting ornamental traits at the University of Tennessee Arboretum, resulting in five released cultivars (‘Empire’, ‘Pam’s Mountain Bouquet’, ‘Red Steeple’, ‘Melissa’s Mountain Snowfall’, ‘Sarah’s Mountain Pixie’; **Supplementary File 2**). Although no cultivars derived from controlled crosses were commercially released from the UTIA breeding program, controlled crosses have been completed for research purposes (Wadl et al., 2009; Wang et al., 2009). One population of flowering dogwood, derived from a honeybee-mediated controlled cross, is still present at the University of Tennessee Arboretum today (Wang et al., 2009).

The oldest and longest-running dogwood breeding program was started in 1965 by Dr. Elwin Orton at Rutgers University (Orton, 1985; Molnar and Capik, 2013). Orton initiated the Rutgers University Woody Ornamentals Breeding Program with the few commercially available pink-red bracted cultivars of *C. florida* forma *rubra* (Orton, 1982). Initially, Dr. Orton’s big-bracted dogwood breeding program focused on hybridizing flowering, kousa, and Pacific dogwoods to improve pink-red bracted cultivars (Orton, 1982). Due to the susceptibility of flowering dogwood to the common dogwood borer​​, as well as the emergence of dogwood anthracnose and powdery mildew, the program was narrowed to breeding solely flowering dogwoods and further intercrossing the few fertile interspecific hybrids that had already been generated by Orton (**Supplementary File 2**; Molnar and Capik, 2013). The resulting hybrids were more vigorous than either flowering or kousa dogwood (Ranney et al., 1995). The Stellar^®^ Series and many other patented hybrids originated from these original F1 interspecific crosses using *C. florida*, *C. nuttallii*, and *C. kousa* individuals (**Supplementary File 2**). A frequently used parent in these early crosses was *C. kousa* K2 (Mattera et al., 2015). Since then, the interspecific hybrids involving all three species have been mostly intercrossed but with occasional backcrossing to pure *C. kousa* depending on the pedigree (see Population-Level Genetics section below for more information). Progeny have repeatedly been selected for kousa-like phenotypes over multiple generations to develop the advanced interspecific hybrids in the breeding program today. These advanced interspecific hybrids have led to the release of cultivars like Scarlet Fire^®^ (Molnar et al., 2017) which largely has signatures of *C. kousa*, with only a few alleles from *C. nuttallii* and *C. florida* (Moreau et al., 2024). Current breeding goals for flowering dogwood include combining pink-red colored bracts, pathogen tolerance, improved bloom displays, and a range of growth architectures from small compact trees to large landscape specimens. For kousa dogwood, the breeding efforts focus on producing more stable and deeper pink bract coloration, larger and unique bract shapes, improved leaf quality and textures, and extended bloom times (Molnar, 2022).

### 4.3 Traditional dogwood propagation

In commercial nursery operations, dogwood trees are most commonly propagated via grafting/budding and through the germination of seeds, and more rarely, using rooted stem cuttings. Tissue culture has been used on a limited scale to support breeding programs (see below). Seed propagation is used to produce both conservation planting and rootstock trees. Dogwood cultivars are commonly propagated by graft insertion between late-July and early-September (Dirr and Heuser, 2006) of a chip bud (scions) into a similarly-shaped cut of a seedling sown the previous year (rootstock) (Halcomb, 2002). Whereas the scion is sourced from a specific cultivar, non-uniform rootstocks originate from wild-collected dogwood seeds. This grafting technique is labor-intensive and does not produce fully clonal plants, as all the rootstocks are genetically different from the scion. Variations in the rootstocks may influence the physical appearance and growth of the grafted bud of the desired cultivar (Migicovsky et al., 2021). For propagation via cuttings, softwood, terminal (branch tip) cuttings of dogwood can be taken in June and July and dipped in rooting hormone. With ideal moisture and drainage, roots develop on 90-100% of flowering dogwood cuttings within 5 to 8 weeks, and about half of kousa dogwood cuttings within 12 weeks (Dirr and Heuser, 2006).

In production, *Cornus* spp. trees are often grown in containers. A well-drained, evenly moist soilless container substrate is critical for limiting the incidence of root rot pathogens (Halcomb, 2002; Fulcher et al., 2012). For the production of trees grown and sold in containers, growers generally transplant bare-rooted seedling liners (Fulcher et al., 2012). Container-grown transplanted liners reach salable size in three to three and a half years (Fulcher et al., 2012). Fertilizer needs throughout both container and field production have also been investigated (Halcomb, 2002; Fulcher et al., 2012). Field-grown *Cornus* spp. flowering trees are transplanted as bare-rooted, and usually grafted, tree liners. Liner planting depth should allow for the tree root flare to be visible once the soil has settled in the dug hole or furrowed trench. Transplanted trees that are planted too deeply are prone to adventitious root formation, limited air availability in the root zone, and increased incidence of root rot pathogens (LeBude and Bilderback, 2008). Field-grown trees that are dug and sold balled-and-burlapped typically require between five and six years to reach salable size (Halcomb, 2002).

### 4.4 *In vitro* propagation (tissue culture)

*In vitro* propagation, or micropropagation, enables rapid, large-scale plant production with genetic uniformity. For many years, *in vitro* clonal mass propagation of big-bracted *Cornus* species has been tried (**Table 1**). The majority of the attempted methods have been optimized using young tissues. There are two primary techniques used for *in vitro* propagation: somatic embryogenesis (SE) and axillary bud proliferation (ABP).

**Table 1:** *In vitro* somatic embryogenesis and shoot proliferation studies of *Cornus florida*, *C. kousa*, and *C. nuttallii*.

Somatic embryogenesis						
Species	Cultivar	Explant	Medium^a^	PGRb^b^	Primary results	Reference
*C. florida*	Wild	12-15-week zygotic embryos	MS/SH	3 or 5 mg 2,4-D + 1 mg KIN/then no PGRs	Direct embryos; only 12% plants- none past first true leaf stage	Trigiano et al., 1989
*C. florida*	Wild	Zygotic embryos	WPM + 2 More	None, Picloram, IBA, 2,4- D	92% conversion to plants in soil; Suspension cultures	Gladfelter and Wilde, 2019
Axillary Bud Proliferation						
Species	Cultivar	Explant	Medium^a^	PGRb^b^	Primary Results	Reference
*C. kousa*	‘Rutpink’ Scarlet Fire^®^ PP28311P3	Axillary buds (presumably)	Knight Hollow Nursery, Middleton, WI	Methodology is proprietary	Proliferating sustainable cultures- whole plants on a commercial scale	Molnar et al., 2017
*C. kousa* ‘Satomi’ × *C. hogkongensis* (*Cornus* ‘NCCH1’)	Hybrid	Apical and axillary buds	Various, but WPM “best”	Shoots: 0.5 µM IAA and 10 μM BAP; Roots: 2.5 μM IAA	Establishment of proliferating shoots and whole plants; 72.5% rooting	Lattier et al., 2014
*C. kousa chinensis*	‘Milky Way’ and *chinensis*	Axillary buds and “shoot material”	BW	Shoots: none; Roots: 1.1 µM NAA and 3.0 µM IBA	Proliferating shoots and whole plants; 100% rooting	Ishimaru et al., 1998
*C. nuttallii*	Wild	Axillary buds from seedlings	MS	Shoots: 0-10 µM BA; Roots: IAA as needed	Whole plants; rooting % not reported	Edson et al., 1997
*C. florida*	Wild	Axillary buds from seedlings	WPM	Shoots: 3.3 µM BA	Whole plants not reported	Declerck, V. and S.S. Korban, 1994
*C. florida*	Wild	Axillary buds from seedlings	WPM	Shoots: 2.2- 4.4 µM BA; Roots: 4.9 µM IBA	Whole plants; 50% rooting	Kaveriappa et al., 1997
*C. florida*	Wild	Axillary buds from seedlings	WPM	Various concentration; best: 4.4 µM IBA then 4.9 µM IBA	Whole plants; 83% rooting	Sharma et al., 2005

^a^Growth Media. MS - Murashige and Skoog (1962). SH - Schenk and Hildebrandt (1972). WPM - Lloyd and BMcCown (1980). BW – Murakami et al. (1998). ^b^Plant Growth Regulators. 2,4-D – 2,4-dichlorophenoxyacetic acid. BA – 3,4-Benzopyrene. IAA – Indoleacetic acid. IBA – Indole-3-butyric acid. KIN – 6-furfurylaminopurine. NAA – 1-naphthylacetic acid. Picloram – 4-amino-3,5,6 trichloropyridine-2-carboxylic acid.

The process of SE for dogwoods typically begins with *in vitro* culture of zygotic embryos (not vegetative tissues) to produce embryo-like structures or somatic embryos that can germinate and develop into plants. SE has successfully been achieved for flowering dogwood using zygotic embryos as starting explant material (Trigiano et al., 1989; Gladfelter and Wilde, 2019). However, big-bracted dogwoods are obligatory outcrossing species and are self-incompatible. Because SE-derived plants are not clones of the maternal parent due to genetic recombination in the zygotic embryo formed via outcrossing, any plant derived from zygotic embryos will probably not display the exact traits of the desired maternal parent. However, SEs from zygotic embryos could potentially provide unlimited copies of unique F_1_ genotypes, which could be useful for research and/or breeding and developing new cultivars. Obligate outcrossing limits the utility of SE from zygotic embryos for large-scale dogwood clonal propagation of distinct cultivars.

Axillary bud proliferation involves culturing axillary buds on nutrient media amended with growth regulators to induce shoot and root formation, thereby producing exact or nearly exact clones of the original plant. For ABP in dogwood, progress has been made in all three popular species and hybrids, and the rooting percentages have increased (**Table 1**) (Declerck and Korban, 1994; Edson et al., 1997; Kaveriappa et al., 1997; Ishimaru et al., 1998; Sharma et al., 2005; Lattier et al., 2014). However, the currently released protocols are optimized for seedlings and are not suitable for clonally propagating mature cultivars. There has been one commercially successful ABP system for cultivars, but the methodology is proprietary (Knight Hollow Nursery, Middleton, WI).

## 5 Genetics studies

### 5.1 Overview of genetic and genomic resources

**Figure 4:**
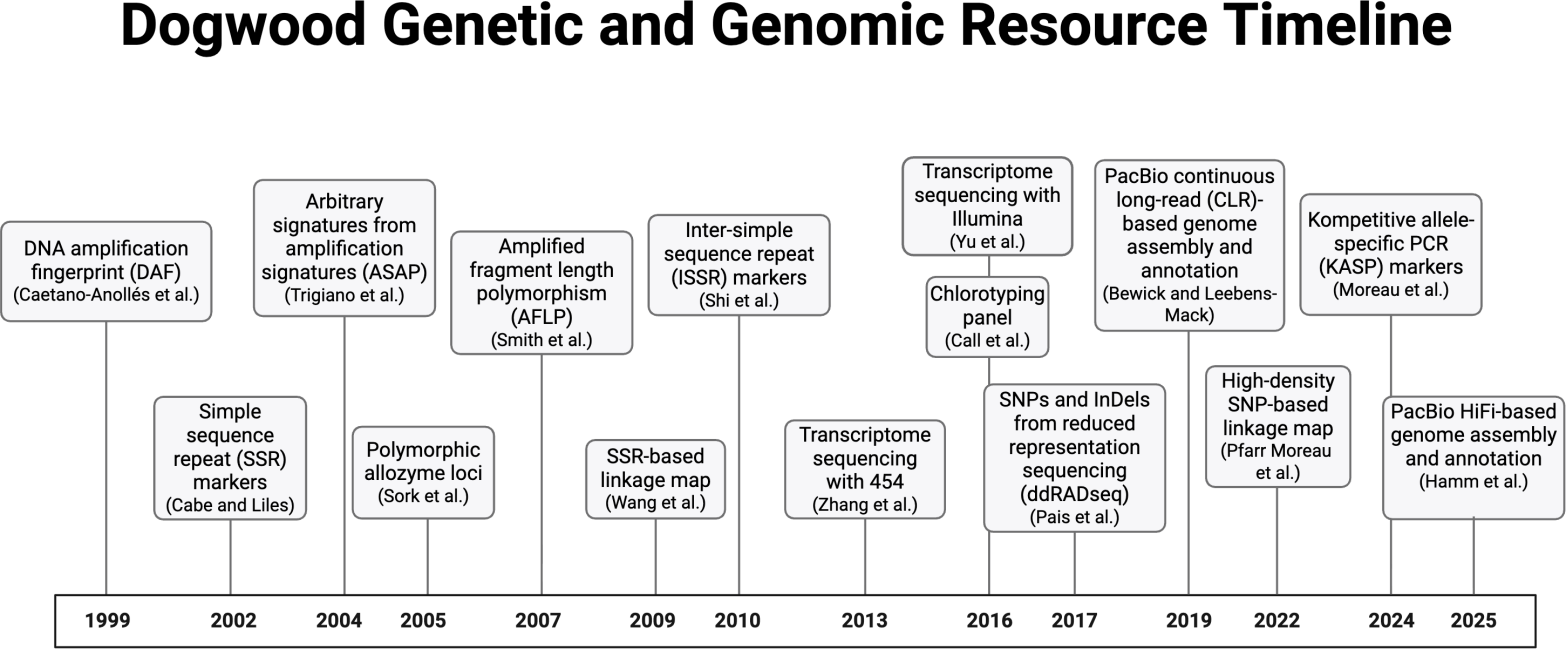
Timeline of dogwood genomic resources (Created in BioRender, 2025).

Most of the early genetic research on dogwood focused on molecular markers, followed by the development of genomic resources, including genomes and transcriptomes (**Figure 4**). The first markers were primarily designed to identify and differentiate individuals and cultivars in breeding programs (described in more detail below). These early markers included arbitrary primers in DNA amplification fingerprinting (DAF) and arbitrary signatures from amplification profiles (ASAP) (Caetano-Anollés et al., 1991, Caetano-Anollés et al., 1999; Trigiano et al., 2004). The markers were expanded to restriction enzyme-dependent types, including amplified fragment length polymorphism (AFLP) (Smith et al., 2007) and chlorotyping panels (Call et al., 2016; Nowicki et al., 2018). Polymorphic allozyme loci have also been developed for natural stands of flowering dogwood (Sork et al., 2005).

Molecular markers then progressed to microsatellites, or simple sequence repeats (SSRs), which require sequencing information and are more reliable than previous markers. Simple sequence repeats were developed for flowering dogwood (Cabe and Liles, 2002; Wang et al., 2008) and kousa dogwood (Wadl et al., 2008a; Nowicki et al., 2020). The information from these markers was also used to develop inter-simple sequence repeat (ISSR) markers (Shi et al., 2010; Yuan et al., 2019) across species (Wadl et al., 2010a; Keir et al., 2011).

With decreasing costs of next-generation sequencing, reduced representation sequencing methods have become widely used for simultaneous marker discovery and genotyping of single-nucleotide polymorphisms (SNPs) and small insertions and deletions (indels) (Andrews et al., 2016). Advantages of these approaches include high marker yield and ease of use regardless of reference genome availability. Variations of reduced representation sequencing have been applied to dogwood, including double-digest restriction-site associated DNA sequencing (ddRAD-seq) (Pais et al., 2017; Moreau et al., 2024). Building on this work, kompetitive allele-specific PCR (KASP) markers were developed based on the SNPs identified using ddRAD-seq to distinguish subspecies of kousa dogwoods (Moreau et al., 2024). These high-throughput genotyping strategies expanded their application from cultivar and individual identification to genetic maps, QTLs, and population genetic studies (see below for details).

Several genomic resources have been developed for dogwoods using next- and third-generation sequencing, including transcriptomes and several genome assemblies. Transcriptomes have been generated for flowering dogwood (Zhang et al., 2013; Yu et al., 2017; Melton et al., 2020) and kousa dogwood (Yu et al., 2017; Melton et al., 2020), primarily as resources for evolutionary biology and functional genomic research. These transcriptomes, assembled using various methods, were based on sequencing data from either Illumina (Yu et al., 2017; Melton et al., 2020) or 454 (Zhang et al., 2013) platforms. Although preliminary, they have been annotated for predicted gene function. In addition, a contig-level reference genome assembly was developed for *C. florida* ‘Appalachian Spring’ (Bewick and Leebens-Mack, 2019). This assembly, generated using PacBio continuous long-read (CLR) technology, includes 4,304 contigs with 83,056 annotated genes. A separate Illumina-based genome assembly is available for kousa dogwood (NCBI BioProject: PRJNA807610), although it remains highly fragmented, comprising nearly 300,000 scaffolds. Recently, updated PacBio High-Fidelity (HiFi) and Hi-C sequencing were used to assemble high-quality chromosome-scale diploid genomes for *C. florida* ‘Appalachian Spring’ and *C. florida* ‘Comco No. 1’ Cherokee Brave™ (Hamm et al., 2025). The four assemblies ranged from 363 to 505 contigs/assembly, 98.0-99.3% complete BUSCOs (genes and proteins), 28,558 to 28,768 genes/assembly, and 96-97% of the genome placed into chromosomes (Hamm et al., 2025). The highly consistent statistics across these four assemblies demonstrate their high quality. There is a relatively low number of contigs, an expected number of annotated genes, and a high percentage of complete BUSCOs and percentage of the genome placed into chromosomes.

### 5.2 Cultivar identification

The published use of the earliest molecular markers of *Cornus* species outside of their initial development is limited. DAF markers that were originally developed for a variety of species other than dogwood (Caetano-Anollés et al., 1991) were then optimized for flowering dogwood in Caetano-Anollés et al. (1999). DAF markers were first used to identify cultivars and parents of F1 progeny (Caetano-Anollés et al., 1999; Ament et al., 2000). Due to their lack of polymorphism, DAF markers were then combined with ASAP markers to identify cultivar synonymy in white-bracted flowering dogwood (Windham and Trigiano, 1998) and pink-bracted kousa dogwoods (Trigiano et al., 2004). AFLP markers developed by Smith et al. (2007) were not used outside of that original study.

SSRs have found a much broader use and have frequently been used to differentiate and identify *Cornus* cultivars and investigate parentage (e.g., Wadl et al., 2008b; Pfarr et al., 2021). They have also been used to prove uniqueness in cultivar releases for both flowering and kousa dogwoods (Molnar et al., 2017; Trigiano et al., 2023). In patents for new dogwood cultivars, SSRs have been used to establish pedigrees, such as the potentially apomictically derived ‘Erica’s Appalachian Sunrise’ (Trigiano and Wadl, 2020).

SNPs and indels from ddRADseq have also been used to identify cultivar synonymy and confirm reported parentage in commercially available cultivars (Moreau et al., 2024). Many of the pink-bracted individuals proved to have ‘Cherokee Chief’ as one of the parents using these markers (Moreau et al., 2024). As has previously been suggested, there is considerable cultivar synonymy in dogwoods, and the data indicate that parents are not always recorded accurately. For example, ‘Prosser’ is recorded as a parent of ‘Cherokee Chief,’ but SNP data suggested that they are genetically identical (Moreau et al., 2024). Considering the self-incompatibility and inbreeding depression of dogwoods, the availability of various molecular markers provides an empirical test for reconstructing pedigrees and identifying synonymy, making it an important tool for current and future breeding and propagation efforts.

### 5.3 Population-level genetics

Molecular markers have also been used to interrogate population-level questions for flowering dogwood, and to a lesser extent, kousa dogwood. Natural populations of both flowering and kousa dogwood have been used to investigate genetic diversity and landscape and genetic connectivity in the context of habitat loss and climate change. Specifically, studies on flowering dogwood have also addressed the impact of dogwood anthracnose and the presence of genetic tolerance.

Chloroplast sequence variability (Call et al., 2016), nuclear SSRs (Hadziabdic et al., 2010, Hadziabdic et al., 2012; Mitchell et al., 2023), and ddRAD-seq-derived SNPs (Pais and Whetten, 2020) have been used to investigate the impact of dogwood anthracnose in natural populations of flowering dogwood. Despite the high pathogen presence and tree mortality across the distribution range, flowering dogwoods have maintained high gene flow and exhibit weak population structure. This genetic diversity may be maintained by long-distance dispersal by migratory birds (Hadziabdic et al., 2010, Hadziabdic et al., 2012; Call et al., 2016). Whereas flowering dogwood is classified as a species of ‘Least Concern’ for most of the U.S., it has been listed as an endangered species in Canada since 2014 (Mitchell et al., 2023). Although the overall genetic diversity of flowering dogwoods in Southern Ontario has aligned with similar studies conducted in the eastern U.S (Hadziabdic et al., 2010, Hadziabdic et al., 2012; Call et al., 2016; Mitchell et al., 2023), this does not appear to be the case specifically in the younger trees (Mitchell et al., 2023). In the Canadian populations investigated, the genetic diversity of younger trees is lower than older trees, and strategies for restoring flowering dogwood populations were recommended (Mitchell et al., 2023).

Reduced representation sequencing methods have been used to investigate loci under selection pressure that may facilitate greater abiotic and biotic resilience in flowering dogwoods. SNPs from ddRAD-seq data were used to identify candidate loci associated with climate, soil properties, and plant health in dogwood anthracnose-infected locations (Pais et al., 2017). The same ddRAD-seq data was used again and supplemented with untargeted metabolite profiling (Pais et al., 2018). This study identified certain SNPs that were previously found to be under selection in Pais et al. (2017) as being associated with an iridoid glucoside, which could play a role in plant defense and tolerance to the pathogen causing dogwood anthracnose (Pais et al., 2018). A geographically broader population was then used for a genome-wide association study (GWAS) to again identify loci that are under selection and associated with ecological and diseased regions (Pais and Whetten, 2020). A lectin-domain receptor kinase identified by Pais et al. (2017) was again found to be under selection, indicating differential allele frequencies between dogwood anthracnose-diseased and non-diseased areas (Pais and Whetten, 2020). This lectin-domain receptor kinase was suggested to be another gene of interest for tolerance to the pathogen causing dogwood anthracnose (Pais and Whetten, 2020). ddRAD-seq data can also be used to identify diseased vs. non-diseased regions, as DNA from *D. destructiva* (the pathogen that causes dogwood anthracnose) can reliably be detected using ddRAD-seq data (Pais and Whetten, 2020).

The developed molecular markers have also been used in pollen flow studies to investigate how disturbances to landscape connectivity affect genetic diversity. Allozymes were used to study the effects of silviculture on pollen movement throughout natural stands of flowering dogwoods in the Ozark Mountains, and it was found that thinning the forest provides greater pollen movement (Sork et al., 2005). SSRs were used to study pollen movement in a natural setting, utilizing landscape genomics, and found that genetic structure is dependent on the spatial heterogeneity of mating individuals and the quality of canopy cover (Dyer et al., 2012). A combination of SSRs and chloroplast sequences was used to forecast the genetic vulnerability of kousa dogwoods in their native range under the threat of climate change, where their distribution is expected to shrink as temperatures increase (Guan et al., 2021).

Outside of traditional population genetics studies investigating gene and pollen flow, as well as identifying loci under selection, general genetic diversity studies have been used to investigate flowering and kousa dogwood. The chlorotyping panel developed by Call et al. (2016) was expanded and used to investigate the chlorotype diversity in cultivated flowering and kousa dogwood (Nowicki et al., 2018). The authors found that the main chlorotypes present in wild flowering and kousa dogwoods are also found in cultivated ones, indicating that cultivars have captured some degree of wild genetic diversity (Nowicki et al., 2018). The genetic diversity of wild-collected kousa dogwoods was investigated using genic and genomic SSRs (Nowicki et al., 2020) and ISSRs (Yuan et al., 2019). Both studies suggested high genetic diversity and included wild-collected (Yuan et al., 2019) and herbaria/arboreta samples (Nowicki et al., 2020). The population structure of kousa dogwood was found to correspond with the country of origin and align with the subspecies differentiation in the discriminant analyses of the principal component, but not in the STRUCTURE analysis (Nowicki et al., 2020). The ISSR markers also indicated mixed levels of differentiation depending on the analysis (Yuan et al., 2019).

Most recently, SNPs and indels from ddRAD-seq were used to investigate the genetic diversity of flowering, kousa, and Pacific dogwood cultivars and wild-collected accessions (Moreau et al., 2024). In accordance with all other diversity studies (Hadziabdic et al., 2010, Hadziabdic et al., 2012; Call et al., 2016; Nowicki et al., 2018, 2020; Pais and Whetten, 2020; Mitchell et al., 2023), high genetic diversity was found. The pink-bracted flowering dogwood trees formed a distinct clade from the white-bracted individuals, with subsp. *urbiniana* as an outgroup. This finding suggests that the same mechanisms control color in all flowering dogwoods with pink-red bracts, but further evaluation is needed. Additionally, the hypothesis that the genes responsible for the newly achieved dark pink color in the bracts of the Rutgers advanced interspecific hybrids are from flowering dogwoods was investigated. However, flowering dogwood DNA introgressions were not reliably detected in the dark pink breeding selections using over 8,000 species-specific markers, although many contained small Pacific dogwood introgressions. With the emerging new genomic resources and additional metabolomics and anthocyanin profiling, the genetic source of the dark pink phenotype and the potential for Pacific dogwood modifier loci could be more thoroughly investigated. Lastly, for kousa dogwoods, in contrast to some previous studies, there was a clear separation between subsp. *chinensis* (from China) and subsp. *kousa* (from Korea and Japan).

### 5.4 Quantitative trait loci

SSRs have been further extended to benefit breeding programs through the development of linkage maps and the identification of QTL, solely for flowering dogwood. The SSR markers developed by Wang et al. (2008) were screened against the parents of a “pseudo-F2” cross of flowering dogwood to identify markers that could be used to build a linkage map (Wang et al., 2009). After crossing Cherokee Brave™ and ‘Appalachian Spring’, due to the dogwoods’ self-incompatibility, two of the F1 trees (97–6 and 97-7) were crossed to generate a “pseudo-F2” population. In total, 255 SSR markers and 94 individuals were used for the linkage map construction. This linkage map and “pseudo-F2” population served as the basis for one QTL study, involving red foliage color (Wadl et al., 2011). The linkage map was then updated using GBS-derived SNPs to investigate red foliage and pink bract color (n = 108; Hamm et al., 2025). This new linkage map has 1,148 markers spanning 2,449 cM across 11 linkage groups. It should be noted that the linkage groups were renamed to correspond with the contigs in Bewick and Leebens-Mack’s genome assembly (2019) and therefore do not match the linkage map produced by Wang et al. (2009).

Using the original SSR-based linkage map, four QTL were identified as associated with red foliage color in seedlings (Wadl et al., 2011). However, the QTL identified depended on the method and time of phenotyping (Wadl et al., 2011). The percentage of variance explained by these loci ranged from 1% to 17%, and this variation depended on both the method and the time of phenotyping. With the updated high-density SNP linkage map, one stable, high-effect QTL was identified on linkage group 9 (Hamm et al., 2025). Within this locus, diagnostic SNPs (accuracy = 100% within population) were identified for the binary presence/absence of leaf and bract color in this population. In combination with RNA-sequencing data and the novel genomics resources, candidate genes were identified (see *RNA-sequencing* section for more information) (Hamm et al., 2025). Further research is needed to identify the postulated modifier loci responsible for the varying levels of pigmentation in the leaves and bracts.

The SSR markers and the linkage map from Wang et al. (2009) were used in two additional mapping populations to identify QTL associated with powdery mildew tolerance (Parikh et al., 2017). The powdery mildew-susceptible cultivar ‘Cherokee Princess’ was independently crossed with a moderately tolerant and a highly tolerant genotype. Because the linkage map was developed for a different cross, only 35 and 29 markers segregated in these populations, respectively. Ultimately, only four QTL with intermediate effects were identified, explaining 9.51% to 13.21% of the phenotypic variance (**Table 2**) (Parikh et al., 2017).

**Table 2:** Identified powdery mildew associated QTL in flowering dogwood. Linkage group conversions based on the two main linkage maps are included.

QTL ID	LG^a^	LG^b^	Position (cM)	LOD	R2 (%)	Population	Markers	Publication
QTL-PM1	LG4	LG9	21	3.8	9.5	CP x R14 n = 147	CF403C, CF622C	Parikh et al., 2017
QTL-PM2	LG5^c^	LG3	76	4.1	10.4	CP x R14 n = 147	CF479B, CF696B	Parikh et al., 2017
QTL-PM3	LG9	LG1	66	5.3	13.2	CP x R14 n = 147	CF763C, CF073A	Parikh et al., 2017
QTL-PM4	LG9	LG1	88	4.5	12.5	CP x M19 n = 147	CF533B, CF305C	Parikh et al., 2017
QTL-PM5	LG5	LG3	43.3	3.3	7.8	P25 × P28 n = 195	799	Pfarr Moreau et al., 2022
QTL-PM6	LG5	LG3	3.78-5.01	11.4	58.9	P25 × P35 n = 83	702 and 686	Pfarr Moreau et al., 2022

a LG number based on Wang et al., 2009 linkage map.

b LG number based on contigs in Bewick and Leebens-Mack, 2020 as presented in Pfarr Moreau et al., 2022.

c Note: this was incorrectly labeled as LG8 in Table 1 of Parikh et al., 2017.

To overcome the low marker coverage, ddRAD-seq-derived SNPs were used in unique mapping populations of flowering dogwood to develop linkage maps and identify QTL associated with powdery mildew tolerance (Pfarr Moreau et al., 2022). A similar pseudo-testcross mapping scheme was used in this study as in Parikh et al. (2017), in which one common parent (breeding accession H4AR15P25) was crossed with two other individuals. Like the map developed by Hamm et al. (2025), this map follows the linkage group naming from Bewick and Leebens-Mack’s genome assembly (2019). From here on, linkage groups will be labeled as in Pfarr Moreau et al. (2022) and Hamm et al. (2025). A major and minor QTL were identified on LG3 in the two mapping populations (Pfarr Moreau et al., 2022). Due to their proximity, it is postulated that the QTL designates the same locus in the two populations and could be a good candidate for marker-assisted selection.

Although these QTL studies have made progress in mapping loci responsible for some of the most important phenotypic traits, additional research is still needed. The main limitations of these previous studies are that they either had too few individuals, low marker coverage, or, particularly for powdery mildew symptoms, variable phenotyping due to the inconsistent nature of natural pathogen pressure. Future studies would benefit from the development of a low-cost and reliable genotyping system, based on sequencing like Diversity Array Technology (DArT) where loci are consistently sequenced unlike GBS. With the recently developed genomic resources, the repeatedly identified locus on LG3 warrants further investigation for powdery mildew tolerance. Additional research with larger populations will also be needed to fine-map and identify specific loci, as the positions of the QTLs cover large portions of those linkage groups. More experiments involving controlled inoculations of powdery mildew (or other pathogens) would also reduce environmental interactions. Alternatively, a genomic selection (GS) approach could shift focus from understanding major effect loci to capturing the whole genome effect if breeding programs were to be significantly expanded (Grattapaglia, 2022). Additional research is also needed to validate the candidate genes in other genetic backgrounds and identify modifier loci controlling the variable pigmentation intensity. The new chromosome-level annotated reference genomes will be instrumental in narrowing down loci controlling these economically important traits and allow for comparative genomics with better-understood systems.

### 5.5 RNA sequencing

Published RNA-seq studies on flowering and kousa dogwoods are limited. In addition to the independent transcriptome assemblies generated by Zhang et al. (2013) and Yu et al. (2017) for evolutionary biology research, Melton et al. (2020) developed transcriptomes to investigate the molecular evolution in eastern Asia and eastern North American species. Notably, these transcriptome studies indicate that the genus *Cornus* has possibly undergone a shared whole-genome duplication (Yu et al., 2017). The whole genome duplication event occurred during the late Cretaceous, coinciding with the initial diversification of *Cornus* and an abrupt rise in sea surface temperatures (Yu et al., 2017).

Traditional RNA-seq studies aimed at identifying differentially expressed genes (DEGs) have historically been lacking for all three dogwood species. To date, the only study to successfully use RNA-seq data this way is in Hamm et al. (2025). Other efforts to identify DEGs have been hindered by the absence of closely-related high-quality annotated reference genomes to map the RNA-seq reads to.

Two projects have deposited RNA sequences into the NCBI Sequence Read Archive (SRA) without conducting downstream analyses, including a dissertation (Pavlovic, 2023) and an NSF-funded The Dogwood Genome Project (Grant #1444567: A Model for Woody Ornamental Genomics). Both studies focused on powdery mildew and identifying tolerance mechanisms in flowering dogwood. As part of the NSF project, seven flowering dogwood cultivars were collected at 11 time points following inoculation with the powdery mildew pathogen. In Pavlovic’s dissertation, samples were collected from the same three cultivars included in the NSF grant study, as well as one additional cultivar, at four time points post-inoculation. To date, neither dataset has been analyzed for DEGs. However, with the new genomic resources of Hamm et al. (2025), such analyses are now feasible. These datasets represent a valuable resource for narrowing down the locus within LG3 associated with tolerance to powdery mildew (Parikh et al., 2017; Pfarr Moreau et al., 2022).

In addition to powdery mildew, RNA-seq has also been used to investigate the genetic underpinnings of color variation in both flowering and kousa dogwoods. This was investigated in two published studies (Hamm et al., 2025; Pavlović et al., 2025). Pavlovic (2025) used bracts of three flowering dogwood and two kousa dogwood cultivars when the color was maximally visible for RNA-seq. Anthocyanin-related genes were identified and used in a time course reverse transcription quantitative PCR (RT-qPCR) to determine how the expression of these specific genes changed over the course of the experiment in the studied cultivars. Whereas the expression of these genes changed throughout the experiment, it was not evident that any could be used for marker-assisted selection for a breeding program. In contrast, Hamm et al. (2025) used a time course RNA-seq experiment to identify DEGs, focusing on a locus associated with leaf and bract color of flowering dogwood. The DEGs helped identify candidate genes responsible for the pigmentation in the leaves and bracts of flowering dogwood. These RNA-seq resources could be used in the future to help validate additional candidate genes with potential roles in regulating leaf and bract color.

## 6 Conclusions and future directions

Despite notable advances in phylogenetics and traditional breeding, many research questions remain unexplored for big-bracted dogwoods. Advancements have historically been hindered by the amount of space and personnel required for visually screening and maintaining breeding populations, forcing breeders to choose between many small full-sibling breeding populations and few large full-sibling research populations. The focus has been on maintaining the many small full-sibling breeding populations to facilitate cultivar development and release. However, it takes at least three years to know if an individual has a trait of interest, and many of the trees are removed from the field over many years from lack of traits of interest. If these individuals could be removed from the populations in the greenhouse, prior to planting in the field, time and resources could be focused on large full-sibling research populations, allowing for additional trait mapping. The hurdle to increasing focus on large full-sibling populations has historically been the absence of high-quality genomic resources to interpret or provide biological context to trait mapping results. The recent assembly of annotated chromosome-scale genomes will enable studies to discover and leverage genome-wide variation in flowering dogwoods with improved read mapping capabilities. Additional genomic resources, including inexpensive, high-throughput, reliable genotyping panels (e.g., DArT sequencing), would facilitate more and higher-resolution QTL mapping and accelerate breeding efforts. Additionally, if significant investment is made into building larger populations and optimizing high-throughput phenotyping pipelines, GS could be used to advance genetic gain instead of individually dissecting traits of interest. For review of the implementation of this strategy compared to marker assisted selection in forest trees see Grattapaglia (2022). Furthermore, existing transcriptome data related to powdery mildew tolerance and pink bract development can now be more fully exploited and understood with well annotated genomes.

Once the next steps toward identifying genes of interest in dogwoods are complete, a broader range of research opportunities will become available. For example, although numerous RNA-seq studies have been conducted, which will be helpful for genome annotation, long-read RNA sequencing methods, such as PacBio Iso-Seq, could be instrumental in annotating gene structure, alternative splicing events, and gene duplications (Zhang et al., 2022). Single-cell RNA sequencing could also provide valuable insights into multiple aspects of dogwood research. This sequencing method has been used in the rubber tree (*Hevea brasiliensis* Mull. Arg) to identify the mechanisms underlying powdery mildew infections at the cellular level (Liang et al., 2023). This system can be applied in dogwood research efforts. Single-cell RNA sequencing could also further elucidate the mechanisms controlling coloration patterns in some bracts, as many individuals display coloration in the veins of the bracts. Once the production of anthocyanins is better understood, their breakdown could be investigated using complementary transcriptome, small RNA, and degradome sequencing. These techniques have provided molecular insights into the fading color of *Malus* crabapple petals (Rong et al., 2023).

With these additional resources, functional genomics research can expand in big-bracted dogwoods. So far, only genes from flowering dogwood have been cloned. The first to be cloned were 28 resistance gene analogs from a powdery mildew-tolerant cultivar, ‘Cherokee Princess’ (Shi et al., 2008). The cloned genes were sequenced and blasted against known resistance genes, and 11 of the 28 were identified as unique. A *TERMINAL FLOWER 1*-like (*TFL1*) gene was also cloned from flowering dogwood (Liu et al., 2016). Overexpression of *CorfloTFL1* in *Arabidopsis thaliana* not only delayed flowering but also restored the normal flowering phenotype in the early-flowering mutant. Other avenues of genetic transformation have been explored. The closest species to big-bracted dogwoods to be successfully transformed is *C. canadensis*, a member of the dwarf clade (Liu et al., 2013). Although *C. canadensis* is a subshrub species, it has been used as a model species for functional genetics in *Cornus* and has been successfully employed for functional characterization of a *TFL1* homolog (Liu et al., 2013, 2019). Further development of these transformation systems will be essential for functionally validating candidate genes identified from other research approaches of big-bracted dogwood research.

Overall, big-bracted dogwood research will greatly benefit from the newly developed annotated genome assemblies for representative species. Genes located in identified QTL can be investigated and used as areas of interest for downstream research. New and already produced RNA-sequencing data can be analyzed. Information gained through these methods could then be functionally validated to elucidate the genetic mechanisms underlying key economic traits of dogwood, including bract color and pathogen resistance. As the genetic underpinnings of traits of interest are greater understood, seedlings for cultivar development can then be screened in the greenhouse. The ability to discard seedlings without traits of interest earlier in the breeding pipeline will greatly decrease the amount of time and resources allocated to maintaining many small full-sibling breeding populations. These freed up time and resources can then be invested into furthering our understanding of traits of interest, ultimately leading to faster releases of desired cultivars to consumers.

## Licenses and Permissions

This manuscript has been authored by UT-Battelle, LLC under Contract No. DE-AC05-00OR22725 with the U.S. Department of Energy. The United States Government retains and the publisher, by accepting the article for publication, acknowledges that the United States Government retains a non-exclusive, paid-up, irrevocable, worldwide license to publish or reproduce the published form of this manuscript, or allow others to do so, for United States Government purposes. The Department of Energy will provide public access to these results of federally sponsored research in accordance with the DOE Public Access Plan (http://energy.gov/downloads/doe-public-access-plan).
