# Supplementary material for: Ornamental origins and genomic frontiers: a review of big-bracted dogwood research
Source: Front Plant Sci. 2026 Jan 30;16:1735902. doi: 10.3389/fpls.2025.1735902 (PMC12901405; doi:10.3389/fpls.2025.1735902)
Supplement: Supplementary file 1 [file Table1.docx]

Supplementary Material

# Supplementary Methods

Occurrence records for *C. kousa* were obtained from the Global Biodiversity Information Facility (GBIF). Occurrences were derived solely from human observations and preserved specimens from Asia (n = 7,113) (doi: 10.15468/DL.SRHFN5) (2025). Records lacking location data (n = 5,459) were removed, yielding 1,654 observations with coordinate information. We then constructed three minimum convex hull polygons with 10 km buffer zones in ArcGIS Pro v3.5.0 (www.esri.com) for these curated occurrences within 500 km of the next nearest observation within China, Japan, and Korea, respectively. Observations greater than 500 km outside each minimum convex hull were either assigned a 10 km circular buffer zone or a custom polygon (as appropriate) to represent allopatric populations. The resulting species range for *C. kousa* was clipped using the 1-km resolution “Deciduous Broadleaf Trees” and “Open Water” products from the EarthEnv 1-km resolution Consensus Land Cover dataset v1.0 (Tuanmu and Jetz, 2014). Finally, the boundaries of all polygons were manually curated to avoid over-extrapolation of the species range in areas without occurrence information and to buffer edge occurrences. We did not discriminate between wild and cultivated specimens due to a lack of data. Thus, we caution that this range map is a simple heuristic to support future surveys of *C. kousa* in the wild.

# Supplementary References

Boggess, S. L., and Trigiano, R. N. (2024). Two new cultivars of *Cornus kousa*: “Melissa’s Mountain Snowfall” and “Sarah’s Mountain Pixie.” *HortScience* 59, 105–107. doi: 10.21273/HORTSCI17489-23

Orton, E. R., and Molnar, T. J. (2005). Exciting developments in the world of *Cornus*., in *Combined Proceedings of the International Plant Propagators Society*, (IPPS; 1998), 322.

Santamour, F. S., and McArdle, A. J. (1985). Cultivar checklists of the large-bracted dogwoods: *Cornus florida*, *C. kousa*, and *C. nuttallii*. *Arboric. Urban For. AUF* 11, 29–36. doi: 10.48044/jauf.1985.008

Thompson, R. S., and Little, E. L. (2011). Flowering dogwood (*Cornus florida*) extent, North America. Available at: https://databasin.org/datasets/13cd781470b641deb5516cb9ce7e3131/ (Accessed June 24, 2025).

Trigiano, R. N., Boggess, S. L., Molnar, T. J., Moreau, E. L. P., and Wadl, P. A. (2024). ‘Erica’s Appalachian Sunrise’: An apomitically derived cultivar from *Cornus florida* ‘Comco No. 1’ Cherokee Brave^TM^. doi: 10.21273/HORTSCI17833-24

Windham, M. T., Graham, E. T., Witte, W. T., Knighten, J. L., and Trigiano, R. N. (1998). *Cornus florida* “Appalachian Spring”: A white flowering dogwood resistant to dogwood anthracnose. 33, 1265–1267. doi: 10.21273/HORTSCI.33.7.1265
